# Explaining cross‐national differences in leaving home

**DOI:** 10.1002/psp.2476

**Published:** 2021-05-13

**Authors:** Lonneke van den Berg, Matthijs Kalmijn, Thomas Leopold

**Affiliations:** ^1^ Department of Sociology Radboud University Nijmegen Netherlands; ^2^ Netherlands Interdisciplinary Demographic Institute‐KNAW University of Groningen Groningen Netherlands; ^3^ Institute of Sociology and Social Psychology University of Cologne Cologne Germany

**Keywords:** Europe, leaving home, transition to adulthood

## Abstract

There are large cross‐national differences in the age of leaving home. The literature offers cultural, economic, and institutional explanations for these differences but has not examined all three explanations in one study. We examine these three explanations using data of the European Social Survey (ESS) from 2002 to 2016, supplemented with year‐specific macro‐level indicators from other data sources. We use a dynamic pseudo‐panel design, allowing us to track the home‐leaving behaviour of cohorts born between 1970 and 1999 in 22 European countries. Our findings show that the three sets of explanations are additive rather than competing, each explaining some of the cross‐national differences in leaving home. The cultural context forms the most important explanation for the cross‐national variation. In total, we explain 80% of cross‐national variation in leaving home. Important predictors are religiosity, individualistic family values, change in youth unemployment, GDP and the net replacement rate.

## 1 INTRODUCTION

Leaving the parental home has long been regarded as one of the main steps in the transition to adulthood (Buchmann & Kriesi, 2011). However, as the transition to adulthood has become more complex, long, and individualised in postmodern society (Beck & Beck‐Gernsheim, 2002; Billari & Liefbroer, 2010), scholars have begun to question whether transitions such as leaving home still mark adulthood. The focus in youth studies has shifted towards the situatedness of transitions and the synergies between cultural and transitional approaches to youth life courses (Wyn, 2014). Whereas the transitions literature focuses on moves towards independence, recent studies speak of moves towards interdependence as young adults nowadays often receive parental support throughout young adulthood (Cuervo & Fu, 2020; Wyn et al., 2012). Parents form a “safety net” and protect their child against the uncertainty that characterises the current young adult life phase (Leccardi, 2005). One of the ways in which they offer support is letting the adult child to live in the parental home.

Although leaving home may no longer mark the transition to adulthood, it still provides information about young adults' living situations. Differences over time and between countries show how living situations depend on the social context in which young people grow up (Heggli et al., 2013; Vogt, 2018; Wyn et al., 2012). Europe offers a striking example of cross‐national variation, as the average age of leaving home varies vastly, particularly between northern and southern countries. For example, while Norwegian women born in the 1970s left home at an average age of 19.2, Italian women left at an average age of 26.9 (Billari & Liefbroer, 2010). Although there has been a general trend towards postponement, large differences in the age of leaving home persist (Buchmann & Kriesi, 2011).

Home‐leaving patterns have important effects over the life course (Buchmann & Kriesi, 2011). On the one hand, early home leaving is associated with weaker parent–child relations later in life (Leopold, 2012; Tosi, 2017) and a higher chance of getting in debt and living in poverty, although often for only a short period (Aassve et al., 2007; Oksanen et al., 2016). On the other hand, long stays in the parental home negatively affect parental savings and assets (Maroto, 2017) and young adults' incomes (Billari & Tabellini, 2010). In late home‐leaving countries, young adults could become overly dependent on their parents (Billari & Tabellini, 2010), and families could become “overburdened” (Settersten, 2007).

How can we explain the large cross‐national differences in the age of leaving home? The literature offers three sets of contextual explanations: cultural, economic and institutional. According to cultural explanations, cross‐national differences in the age of leaving home are historically rooted in ‘weak’ and ‘strong’ family systems (Reher, 1998), differences in religiosity (Iacovou, 2002), and an ideational shift during the Second Demographic Transition (SDT) (Lesthaeghe, 2014, 2007; Lesthaeghe & Surkyn, 1988; Van De Kaa, 1987). Economic explanations focus on economic characteristics of a country that influence the opportunities young people enjoy and the constraints they face regarding leaving home, such as economic development and youth unemployment. Institutional explanations focus on differences in the size of the welfare state that affect the degree to which independent living is possible without income or support from parents. These three sets of explanations, sometimes called the ‘welfare mix’ (Vogel, 2002), are often discussed as separate or competing explanations. However, they might complement and interact with one another.

We test macro‐level cultural, economic, and institutional explanations for cross‐national differences in the timing of leaving home using data from the European Social Survey (ESS) from 2002 to 2016 for 22 countries. We have supplemented these data with a rich set of year‐specific macro‐level indicators from various data sources, such as the World Bank, the European Values Study (EVS), the World Values Study (WVS) and the OECD. Our first goal is to examine the strength of the three different sets of explanations. Our second goal is to examine whether the strength of the different explanations depends on other contextual characteristics by estimating interactions between the different contextual explanations. These interactions allows us to examine whether poor economic conditions prolong the stay in the parental home only in countries with strong family ties or countries with little institutional support.

We make two main contributions to the literature. First, we offer one of the first comprehensive studies of macro‐level explanations for cross‐national differences in leaving home. Although theoretical work on these explanations is abundant, empirical work evaluating them is scarce. A few studies have taken the first steps towards understanding cross‐national differences in leaving home by showing correlations between macro‐level factors such as the unemployment rate and the age of leaving home or co‐residence rate (Billari, 2004; Chiuri & Boca, 2010; Iacovou, 2002; Lennartz et al., 2015). However, they have not systematically examined multiple explanations in a single analysis. By testing multiple different sets of explanations, our study contributes to the debate about cultural and structural explanations in demographic research.

Second, we developed a pseudo‐panel design to predict macro‐level year‐specific home‐leaving rates with year‐specific macro‐level indicators. This design has important advantages over the most obvious alternative, a multilevel design with the co‐residence rate as the outcome. First, a multilevel design based on cross‐sectional data would lead to endogeneity problems because it is not possible to determine, for example, whether individuals became unemployed before or after leaving home. Our pseudo‐panel data approach circumvents these problems by using macro‐level predictors that are measured before the home‐leaving rate. Second, a pseudo‐panel approach allows a large number of countries and cohorts to be tracked over time (Pelzer et al., 2005). Our design captures the exact timing of leaving home, whereas the co‐residence rate does not accurately reflect home leaving in a specific year. For example, the co‐residence rate among 30‐year‐olds might be the result of home leaving among young adults from that cohort at age 18 instead of recent macro‐level characteristics. Third, our pseudo‐panel design fits with the goal of our research—examining the extent to which macro‐level characteristics predict leaving home at the macro‐level. Our design also has advantages over the use of international panel datasets containing items on this topic, such as the Generations and Gender Survey (GGS) (Gauthier et al., 2018). These panel datasets are limited in the number of countries and period in which they are conducted. We examine leaving home in 22 countries during a unique period of 15 years that includes time before, during, and after the Great Recession.

## 2 THEORETICAL BACKGROUND

### 2.1 Previous research

A large body of research has described cross‐national differences in the age of leaving home or co‐residence rates in Europe (Aassve et al., 2002; Aassve, Cottini, & Vitali, 2013; Albertini & Kohli, 2013; Billari & Liefbroer, 2010; Billari et al., 2001; Chiuri & Boca, 2010; Iacovou, 2002; Lennartz et al., 2015; Schwanitz & Mulder, 2015). Late home leaving in the south is seen as the prime example of the southern pattern of transition to adulthood, dubbed the “latest‐late pattern” (Billari, 2004; Leccardi, 2005). In Northern Europe, there is a pattern of early home leaving, dubbed the ‘earliest‐early’ pattern. However, a geographical distinction in home‐leaving patterns does not fit for all countries. For example, the age of leaving home in Continental and Eastern Europe varies (Billari & Liefbroer, 2010).

Even though there is ample evidence for contextual differences in the age of leaving home, to our knowledge, only four studies have investigated correlations between macro‐level factors and cross‐national differences in the age of leaving home (Billari, 2004; Chiuri & Boca, 2010; Iacovou, 2002; Lennartz et al., 2015). An overview of these studies and the factors they explored can be found in Table 1. These studies have shown correlations between economic factors and the age of leaving home or co‐residence rate. Moreover, they indicate that the percentage of religious individuals in a country and welfare characteristics such as social expenditure correlate with the age of leaving home.

**TABLE 1:** Overview comparative studies on cross‐national home leaving differences that examined macro‐level explanatory variables

Study	Data	Years/cohorts	N	Method	Dependent variable	Macro‐level explanatory variables	Direction
(Billari, 2004)	Fertility and Family Surveys (FFS)	1960s	19 countries	Correlations	Median age of leaving home	Poverty risk	−
						Pension	+
						Elderly adults in institutions	−
						Suicide rate	−
						Wages replacement rate	−
						Number of associations individuals belong to	−
(Chiuri & Boca, 2010)	ECHP	1994–2001	14 countries, 4240 individuals	Fixed (country) effects logit model	Co‐residence, 18–34 year olds	Unemployment rate	+
						Sexratio	+
						Downpayment	+
						Youth policies	−
(Iacovou, 2002)	ECHP, PSID	1994	13 countries, 67,874 individuals	Scatter plot per variable	Age at which 50% left home	% Catholic/Orthodox	+
						Social expenditure	−
						% Renting	−
						% Youth in insecure employment	No
(Lennartz et al., 2015)	EU‐SILC	2007; 2012	14 countries, 270,000 individuals	Correlations	Co‐residence rate change 2007–2012, 18–34 year olds	Change in employment rate young adults	No
						Change in education enrolment	−
						Housing allowance among renters	No
						Change house‐prices	No
						Mortgage debt to GDP 2007	No

### 2.2 Culture and structure

We examine cultural, economic, and institutional explanations for cross‐national differences in the timing of leaving home. The explanations studied are part of a larger debate about the role of structure (institutions and economy) and culture in demographic behaviour. The assumption behind structural explanations is that human behaviour is guided by a rational decision‐making process in which the benefits of a decision are weighed against the costs. According to this model, the context shapes the opportunities and constraints on which young adults base their decision to leave the parental home. For example, Mills and Blossfeld (2003) offer a model in which globalisation results in increasing uncertainty, affecting young adults' rational decision‐making processes for specific demographic outcomes such as union formation and the choice between marriage and cohabitation.

In contrast to the rational action model, cultural explanations assume that human behaviour is not merely a decision based on opportunities and constraints but is guided by a system of values and norms (Pollak & Watkins, 1993). Values reflect individuals' beliefs about what they find important in life, whereas norms govern how people behave (Schwartz, 2012). Europe is theorised to be divided into a more individualistic north, with a weak family system that prioritises individualistic values, and a more familialistic south, with a strong family system that prioritises collectivist values (Reher, 1998; Viazzo, 2010). These cross‐national differences in values and norms may result in cross‐national differences in behaviour. For example, a recent study shows that cross‐national differences in non‐kin support can be partially explained by differences in individualistic values and familialistic norms (Conkova et al., 2018). Despite a more prominent role of culture in works on family systems (Reher, 1998) and on the SDT (Lesthaeghe, 1983; Van De Kaa, 1987), culture is still often ‘backgrounded’ in demographic research (Bachrach, 2014).

For each type of explanation, we develop specific measures and test their effects in multivariate empirical analyses. By studying cultural and structural explanations both simultaneously and separately, we examine whether cultural characteristics explain home leaving even if we account for economic and institutional characteristics. Accounting for other characteristics is important because economic conditions correlate with cultural characteristics and welfare state provisions have a cultural base (Mayer, 2001). Two caveats must be mentioned regarding our approach to examining cultural and structural explanations. First, the selected predictors could have compositional and contextual effects on home leaving. For example, the effect of the youth unemployment rate on the home‐leaving rate could be the result of an individual effect of unemployment on home leaving, but it could also represent a contextual effect if employed people postpone home leaving when the unemployment rate is high. Second, although we categorise and discuss the characteristics based on whether they could be considered primarily cultural, economic, or institutional, these categories are not mutually exclusive. For example, housing prices have both institutional and economic components. Our main focus is on the extent to which each of the specific explanations accounts for cross‐national differences in leaving home rather than on the category to which it belongs.

### 2.3 Cultural explanations

We first explore cultural characteristics of societies to explain cross‐national differences in leaving home. One prominent cultural explanation is systematic variation in family systems along geographical lines. Reher (1998) has argued that a north–south gradient divides Europe into countries with a weak family system (centre and northern countries) and countries with a strong family system (the southern countries) based on religious traditions. Weak family systems are characterised as systems in which the individual takes precedence over the family, autonomy and independence are valued, and intergenerational ties are weaker. Strong family systems are systems in which the individual develops within the family, the family takes precedence over the individual, and intergenerational ties are strong. According to Reher (1998), early home leaving in the north and late home leaving in the south is a prime example of differences between the two regions in intergenerational support and values. Recent evidence suggests that the division into weak and strong family systems also applies to a division between the west and east, with stronger family norms in the east than in the west (Daatland et al., 2011). This fits with the Hajnal (1965) line that runs from Trieste to St. Peterburg and divides countries based on marriage patterns.

A second cultural explanation comes from the literature on the SDT. The SDT holds that the decrease in fertility, the increase in non‐marital cohabitation, and the increase in divorce are connected and could be explained by an ideational shift (Lesthaeghe, 1983, 2007; Lesthaeghe & Surkyn, 1988; Van De Kaa, 1987). This ideational shift entails a shift from more altruistic to more individualistic norms and attitudes and secularisation as a manifestation of individual autonomy. The timing and speed of these changes differ between countries (Van De Kaa, 1987). Several scholars have made connections between the SDT and leaving home or the transition to adulthood more generally (Billari et al., 2001; Buchmann & Kriesi, 2011; Mulder et al., 2002). They argue that more individualistic values have led to a stronger desire for autonomy and made it more acceptable for young adults to leave home at a young age.

Only a few previous studies have examined cultural explanations for cross‐national differences in leaving home. Iacovou (2002) shows that a higher percentage of Catholics in a country is associated with later home leaving. Research on individual norms regarding the age of leaving home shows that late home leaving is more accepted by people in southern and eastern regions of Europe than by people from northern and western regions and that these differences in norms are partially explained by the proportion of people in the region that belong to a religion (Aassve, Arpino, & Billari, 2013).

In our analyses, we compare country‐periods based on religiosity, family values, and parental responsibilities. We define country‐periods in which people are less religious, have more individualistic values, and ascribe fewer responsibilities to parents as *individualistic* and country‐periods in which people are more religious, less individualistic, and ascribe more responsibilities to parents as *familialistic*. Our first hypothesis is as follows: *Home‐leaving rates are higher in country‐periods that are more individualistic than in country‐periods that are more familialistic (Hypothesis 1)*.

### 2.4 Economic explanations

Second, we examine economic differences between countries to explain cross‐national differences in the home‐leaving rate. Economic characteristics are the most dynamic of the three sets of characteristics and fluctuate in cycles over time. They changed substantially during the Great Recession of 2007 to 2011, which especially affected younger people (Aassve, Cottini, & Vitali, 2013). We consider the state of the economy in a specific year and country rather than a general year‐effect of the recession for two reasons. First, the timing and impact of the recession differed across countries. For example, between 2007 and 2012, the youth unemployment rate fell by 3% in Germany, while it increased by more than 30% in Spain and Greece (Aassve, Cottini, & Vitali, 2013). Second, although the recession substantially worsened economic conditions, those conditions find their roots in a trend towards greater insecurity that started in the 1970s and 1980s (Furlong et al., 2017).

w?>Young adults negotiate economic risks in the young adult life phase (Furlong et al., 2017; Furlong & Cartmel, 2006). Poor economic conditions do not only affect young adults who are unemployed or who have a low income, but also increase uncertainty about economic prospects among all young adults (Blossfeld et al., 2006; Buchmann & Kriesi, 2011; Oppenheimer, 1988). The increasing insecurity in the labour market that is now part of young adulthood requires young people to build their lives in new ways (Furlong et al., 2017). Young adults might stay in the parental home until they have more information about their prospects and are in a more stable economic position.

The housing market could also be an important factor in the decision to leave the parental home. Young adults who have trouble finding suitable housing due to high housing prices may postpone their move out of the parental home. The age of leaving home differs between housing regimes (Mulder & Billari, 2010). Home leaving is late in countries with ‘difficult’ homeownership regimes, characterised by a high prevalence of homeownership and limited availability of mortgages.

Previous research has found mixed effects of (youth) unemployment on the age of leaving home. Whereas one study found no effect of the change in the unemployment rate (Lennartz et al., 2015), another study found a negative association between the (youth) unemployment rate and leaving home (Chiuri & Boca, 2010). An explanation for these mixed findings could be that Lennartz et al. (2015) examined changes in unemployment and co‐residence rates rather than the absolute rates. Late home leaving is more common in countries with a higher risk of poverty (Billari, 2004), lower youth incomes (Billari, 2004), more owner‐occupied housing (Iacovou, 2002), and a high down payment rate (Chiuri & Boca, 2010).

Based on these considerations, our second hypothesis is as follows: *Home‐leaving rates are higher in country‐periods with better economic conditions than in country‐periods with poorer economic conditions (Hypothesis 2)*.

### 2.5 Institutional explanations

Lastly, we explore institutional characteristics to explain differences in leaving home between countries and over time. We focus on welfare state regulations, the provisions offered by the state to protect and promote the well‐being of its citizens. Esping‐Andersen (1999, 2006) has distinguished three welfare regimes—Social‐Democratic regimes, Conservative regimes, and Liberal regimes—and shown that family formation patterns differ between these regimes. We do not examine welfare regimes but consider two more specific indicators of welfare state provisions. This allows us to account for differences in welfare state provisions between countries from the same welfare regime and for differences in welfare state provisions over time.

The welfare state might provide young adults with financial means to live independently of their parents and function as a “safety net” that limits some of their economic uncertainties. The knowledge that the welfare state could step in and support them when they lose their job or are in need of financial support for other reasons could ease young people's uncertainty about leaving home. Family can be a source of support if there is little institutional support (Cuervo & Fu, 2020). One way in which the family offers support is by letting the child live in the parental home if a lack of institutional support makes independent living more difficult.

Patterns of early transitions in the north of Europe and late transitions in the south are often linked to differences in welfare state provisions. For example, early home leaving and a lack of planning among Norwegian youth are perceived to be the result of confidence that the welfare state can take care of them (Heggli et al., 2013). Several studies have found more direct evidence for the importance of institutional factors for leaving home. Higher general social expenditure (Iacovou, 2002), youth social expenditure (Chiuri & Boca, 2010), and unemployment benefits (Billari, 2004) are associated with earlier home leaving. Recent research on the impact of changes in student loan provisions in the Netherlands has shown that cuts in financial support for students have led to postponed home leaving (Van den Berg, 2020). This leads to our third hypothesis: *Home‐leaving rates are higher in country‐periods with a larger welfare state than in country‐periods with a smaller welfare state (Hypothesis 3)*.

### 2.6 Interactions between macro‐level factors

Poor economic conditions might not have the same impact on leaving home in all countries. The family (culture) or the state (institutions) could protect young adults from economic hardship.

The family forms one of the main institutions that people turn to in times of economic difficulties (Leccardi, 2005; Settersten, 2007). Whether poor economic conditions promote intergenerational co‐residence could depend on cultural norms about whether the family is regarded as the primary safety net. According to Reher (1998), the family functions as a form of protection against social and economic difficulties, especially in strong family systems. In these countries, young adults might be more likely to consider prolonging their stay in the parental home as a response to economic hardship and uncertainty. Moreover, during economic downturns, parents in countries with stronger family systems might more often feel a responsibility to let their children stay with them, whereas parents in more individualistic countries might more often support the child financially (Albertini et al., 2007).

Next to family, the welfare state forms one of the primary institutions that is called upon in times of need. An extensive welfare state offers young adults economic resources for independent living regardless of the economic conditions in the country and offers protection against risks (Settersten, 2007). Hence, compared to young adults in countries with few welfare state provisions, young adults in more extensive welfare states would be expected to experience less uncertainty and economic hardship during economic downturns, lessening the need for delaying home leaving.

Previous research has not examined interactions between macro‐level economic, cultural, and institutional characteristics. However, several studies have shown that economic characteristics measured on the micro‐level have a stronger impact on leaving home in Southern Europe (Aassve et al., 2002, 2007; Albertini & Kohli, 2013; Arundel & Lennartz, 2017). This might be due to stronger family ties or a smaller welfare state in these countries. A study comparing three countries—the United States, Germany, and the Netherlands—showed that employment mattered less in countries with a stronger welfare state (Mulder et al., 2002).

These considerations lead to the following hypothesis regarding economic and cultural characteristics: *Economic characteristics have a stronger effect on the home‐leaving rate in more familialistic country‐periods than in more individualistic country‐periods (Hypothesis 4)*. The expectation regarding economic and institutional characteristics is the following: *Economic characteristics have a stronger effect on the home‐leaving rate in country‐periods with a smaller welfare state than in country‐periods with a larger welfare state (Hypothesis 5)*.

## 3 METHOD

### 3.1 Data

We use a pseudo‐panel design that simulates a panel data structure with repeated cross‐sectional data. We use data from young adults aged 16 to 34 who participated in the ESS. The ESS rounds are 2 years apart and cover a 15‐year period, from 2002 to 2016. There are several advantages of using data of the ESS for our purposes. First, the ESS data are recent and cover the time before and after the Great Recession. We are able to track birth cohorts up to 1999 that have not yet been studied in cross‐national research on leaving home. Second, left‐censoring is minimised as these data allow us to model home leaving from age 15 onwards. Third, the representative cross‐sectional samples of the ESS are high‐quality. We combine these data with aggregated country‐ and year‐specific data from the OECD, the World Bank, the EVS and the WVS.

Our unit of analysis is not the individual, but groups of at least 10 individuals from a specific country, cohort, and gender (CCG groups). On average, these groups consist of 27.5 individuals. We study these groups over time as the cohorts age over ESS rounds. Using repeated observations for these units across ESS rounds, we create a CCG by calendar year file (CCGY).

### 3.2 Measures

#### 3.2.1 Dependent variable

Our dependent variable is the relative home‐leaving rate. We determine whether a young adult has left home based on the question: ‘Including yourself, how many people – including children – live here regularly as members of this household?’ Students who live away at college do not belong to the parental household (The ESS Sampling Expert Panel, 2016). We consider young adults as having left home if they do not live in the same household as their parents.

The first step in determining the relative home‐leaving rate is estimating the co‐residence rate per CCG group in a specific year. To calculate the co‐residence rate of a group, we estimate the percentage of young adults in a CCG group who live with their parents, using the design weights of the ESS. Next, we estimate the relative home‐leaving rate by dividing the change in co‐residence of a CCG group between the current and the previous wave of the ESS by the co‐residence rate in the previous wave (2 years before). We look at the relative rather than the absolute change in the co‐residence rate because the absolute change is strongly dependent on the previous co‐residence rate and might not reflect the actual home‐leaving behaviour between two waves. The relative home‐leaving rate is more similar to life‐table and logistic event‐history models, which are often used in micro‐level studies on leaving home.

The following example shows how the relative home‐leaving rate is calculated. In Germany in 2004, 14% of women and 29% of men born in 1979 were living with their parents. In 2006, the co‐residence rates of these young adults were reduced to 8% among women and 23% among men, whereas both men and women have an absolute change score of 6, the relative home‐leaving rate is 43% for women and 21% for men. The relative rate is preferable here because it represents the higher chance among women who live in the parental home to leave home between the two waves. As our measure shows, 43% of the women compared to 21% of the men living in the parental home moved out. The calculations are as follows:

Relative home leaving rate: $\mathrm{Yi}=\frac{\left(\text{YiAge}27-\text{YiAge}25\right)}{\text{YiAge}25}*-100$

Example women $\mathrm{Yi}=\frac{\left(8-14\right)}{14}*-100=43\%$

Example men $\;\mathrm{Yi}=\frac{\left(23-29\right)}{29}*-100=21\%$

Our central measure is the change in the co‐residence rate of a birth cohort over time. Changes in the co‐residence rate are produced not only by home leavers, but also by home returners. When it comes to how and why what we study matters, leaving and returning home are two sides of the same coin. Theoretically, leaving and returning are driven by similar mechanisms like the attractiveness of an independent home vis‐à‐vis the parental home (e.g., Arundel & Lennartz, 2017; Stone et al., 2013). This means that given the overall picture that we want to explain, the measure is meaningful, despite being a composite measure. Empirically, there is also overlap. Analyses with the ESS round 9 data^i^ show that cross‐national differences in the percentages of home leavers who again live in the parental home, i.e., returners, closely follow the cross‐national differences in leaving home (Table A1). For example, whereas only 5% of the young adults from Finland aged who have ever left home are living in the parental home at the time of the survey, this was the case for 25% of the young adults from Italy and Slovenia. Finally, it is important to emphasise that the percentage of home returners is low. In round 9 of the ESS, only 13% of the young adults who were living in the parental home had previously lived independently. This means that even though the change in the co‐residence rate is a composite, it is dominated by leaving home, not by returning home.

#### 3.2.2 Independent variables

We derive the independent variables from aggregated country‐ and year‐specific data from the full samples of the ESS, the OECD, the World Bank, the EVS, and the WVS. We standardise and lag all independent variables in the analyses. We use independent variables that are lagged by two years to circumvent endogeneity issues, as non‐lagged variables could explain home leaving by indicators measured after home leaving took place rather than before. We use design weights for the data of the ESS, the EVS, and the WVS. An overview of the values of all independent variables per country and in total can be found in Table 2. Note that this table shows the unstandardized versions of the variables.

**TABLE 2:** Descriptive statistics on the independent variables

	Age	Year	Gender (ref. female)	Religios.	Resp. parents	Ind. values	GDP	GDP change	Youth unemp.	Youth unemp.change	House price	House price change	Social expend. (% GDP)	NRR	N
Austria	25.96	2008.09	0.52	−0.10	0.61	−0.16	37184.98	10811.09	7.53	0.44	94.48	−4.74	26.58	55.36	46
Switzerland	25.70	2010.07	0.50	−0.45	0.74	−0.36	40633.92	5724.25	19.86	1.89	98.41	5.48	26.83	69.27	121
Belgium	25.14	2010.10	0.56	−0.13	0.75	0.68	67434.98	9183.06	7.87	1.26	92.33	4.53	18.21	67.89	102
Czech	26.22	2011.88	0.51	−0.86	0.52	−0.81	18966.22	1484.66	16.09	−0.28	103.17	−6.48	19.28	48.89	81
Germany	25.54	2010.03	0.52	−0.28	0.60	−0.43	39489.71	5402.95	10.57	0.22	104.20	−1.28	25.22	54.41	118
Denmark	23.44	2008.18	0.57	−0.41	0.69	1.85	52416.21	7448.90	10.25	1.88	97.78	−2.84	26.50	82.38	68
Estonia	25.52	2011.04	0.52	−0.62	0.66	−0.76	15349.05	2459.39	19.88	−0.95	139.12	12.14	15.27	34.51	102
Spain	25.93	2008.95	0.50	−0.30	0.83	0.41	27540.62	4020.44	27.37	5.25	92.01	5.72	22.30	47.30	107
Finland	23.59	2009.95	0.58	−0.25	0.58	0.93	43032.59	5497.71	22.16	0.37	91.89	4.71	25.78	65.41	83
France	25.38	2009.77	0.52	−0.51	0.80	0.48	37487.78	5302.39	20.23	0.13	91.99	7.17	29.46	59.58	96
Great‐Britain	25.30	2009.92	0.51	−0.42	0.80	−0.11	41131.89	4688.62	15.63	1.62	97.50	7.25	20.72	56.08	105
Greece	26.00	2007.00	0.50	0.52	0.73	−1.16	23053.80	10019.82	25.04	−4.07	104.98	−7.25	20.30	17.30	36
Hungary	26.45	2009.09	0.49	−0.36	0.75	−0.67	11756.29	2167.31	18.90	1.83	124.58	−18.43	22.05	32.56	94
Ireland	25.62	2012.07	0.49	0.11	0.72	−0.11	53802.31	1581.05	19.81	3.77	102.10	0.34	19.27	73.72	85
Italy	27.20	2004.00	0.47	0.24	0.75	−0.99	22196.51	1571.92	27.73	1.14	83.17	16.21	23.31	2.80	15
The Netherlands	24.55	2009.59	0.53	−0.36	0.80	0.56	45250.30	7181.09	7.68	1.64	96.61	−0.69	20.70	72.43	87
Norway	24.13	2010.16	0.54	−0.42	0.86	1.17	81061.41	9435.09	9.79	−0.51	94.47	6.16	21.25	61.60	90
Poland	25.94	2010.03	0.50	0.60	0.75	−0.62	10746.75	1392.36	30.08	−0.38	111.83	−26.01	20.46	44.12	125
Portugal	26.00	2008.65	0.51	0.07	0.85	−0.02	19724.91	3031.23	16.76	4.25	106.46	−6.27	22.41	50.75	101
Sweden	23.52	2010.29	0.57	−0.61	0.87	0.88	49896.74	6643.52	20.87	1.24	91.59	8.35	26.72	67.87	83
Slovenia	25.84	2009.85	0.49	−0.17	0.79	0.10	20862.48	3002.50	15.46	0.59	115.40	−19.95	22.08	52.64	116
Slovakia	26.94	2008.88	0.50	0.20	0.63	−0.91	14511.34	2475.77	28.13	−2.71	86.54	67.36	16.25	31.61	64
Mean	25.42	2009.82	0.52	−0.23	0.74	0.49	35497.19	5186.31	17.61	1.08	102.42	0.16	22.14	55.18	
Min	18	2004		−0.90	0.48	−1.16	5196.93	−12586.01	4.37	−15.11	49.36	−56.26	12.55	2.80	
Max	34	2016		0.70	0.99	1.85	101668.20	23505.95	46.43	19.77	177.43	148.37	31.94	85.00	
SD	4.96	3.91		0.35	0.10	0.70	20172.19	6943.31	8.35	4.92	21.23	24.23	4.01	15.15	

*Note*: Note that these are the unstandardized version. In the analyses, all independent variables are standardised.

We use three measures for macro‐level cultural characteristics. Religiosity is our first indicator and is measured in the ESS by four items: ‘Do you belong to a religion or denomination?’, ‘How religious are you?’, ‘How often do you attend religious services apart from special occasions?’, ‘How often do you pray apart from religious services?’. Index scores on the individual level of the whole sample (alpha = 0.85) are aggregated to the level of country‐years. Individualistic family values is included based on a set of items in the ESS about gender and family values. These are the following seven items: ‘Women should be prepared to cut down on paid work’, ‘Men should have more right to a job than women’, ‘Approve if person chooses never to have children’, ‘Approve if person chooses to live with partner not married to’, ‘Approve if person chooses to have child with partner not married to’, ‘Approve if person chooses to have full‐time job while having children younger than 3’, ‘Approve if person gets divorced while children are younger than 12’. These items were not included in the same waves. To deal with this item non‐response, we first calculate country‐mean scores per item based on the whole sample, and next combine these country‐mean scores into an index, that is, a personal mean score method (alpha = 0.94). The use of personal mean score has been shown to produce negligible bias (Peyre et al., 2011). Responsibility of parents is based on the following items from the WVS and EVS: ‘With which of the following statements do you agree’: ‘Parents' duty is to do their best for their children even at the expense of their own well‐being’, ‘Parents have a life of their own and should not be asked to sacrifice their own well‐being for the sake of their children’, ‘Neither’. We recode this variable into a dichotomous variable that measures whether people agree with the statement that parents' duty is to do their best for their children even at expense of their own well‐being. We aggregate this measure to the country‐year level. As the year in which the EVS, the WVS, and the ESS were conducted differ and not every country participated in every wave of the EVS and WVS, some of the data for the parental responsibility variable are missing. We reduce the number of missings on this item by estimating the values based on data from other years using interpolation techniques.^ii^

We include six economic indicators. The GDP per capita, change in GDP, the unemployment rate, and change in the unemployment rate are derived from the World Bank. GDP is measured in US dollars. Youth unemployment is measured as the percentage of unemployed youth aged 15 to 24 of the total labour force population of 15‐ to‐24‐year‐olds. We do not only look at the absolute levels of GDP and youth unemployment, but also at the change in these levels between two waves. Housing prices and change in housing prices are derived from the OECD. These indicators reflect the real housing price, which is the ratio of nominal housing prices to the consumers' expenditure deflator.

The institutional indicators are derived from the OECD. The first institutional indicator is the total net social spending, measured by the total social spending as a percentage of the GDP. The second institutional indicator is the net replacement rate (NRR), the percentage of the income that is maintained after unemployment. We use the average NRR for single individuals in the first 5 years of unemployment. This is the income after tax and including unemployment benefits, social assistance, family and housing benefits in the 60th month of benefit receipt.

We control for several group characteristics. These are the year of observation, age, age‐squared, and gender. Both age and age squared are centred.

### 3.3 Sample

Our pseudo‐panel data consists of 2585 CCGY groups which are observed in two consecutive waves. We only keep groups which are observed in at least two waves, because we need information on co‐residence in two waves in order to estimate home leaving between these waves. We keep groups in which the co‐residence rate is at least 5% in the year before (*n = 2334*), as the relative home‐leaving rate could otherwise be too sensitive to small sample differences between two waves. Next, we restrict our sample to groups in which information on all the independent variables is available (*n = 1983*). Then we remove outlier CCGY groups from our sample which show a relative home‐leaving rate of less than −100 (*n = 22*), which would indicate that the co‐residence rate more than doubled between two waves. In a last step, we exclude observations in Ireland between 2002 and 2006 (*n = 36*).^iii^ This leads to a final sample of 1925 CCGY groups (127 CCG groups and 22 countries). The sample selection process is detailed in Table A2. Table A3 shows the number of CCGs included per year.

### 3.4 Analytical strategy

We use a pseudo‐panel design to estimate the relative home‐leaving rate. We study the home‐leaving rate in a linear regression multilevel design, a random intercept model with CCGY groups nested in countries and with year fixed effects. We run models separately with only the cultural, economic, and institutional factors to test Hypotheses 1 to 3. Next, we estimate the full model including all factors to estimate how much all factors combined explain of cross‐national differences in leaving home. Moreover, this model shows whether certain factors explain the effects of other factors. Next, we estimate interactions between the cultural and economic factors to test Hypothesis 4, and between the institutional and economic factors to test Hypothesis 5.

## 4 RESULTS

### 4.1 Descriptive results

**FIGURE 1:**
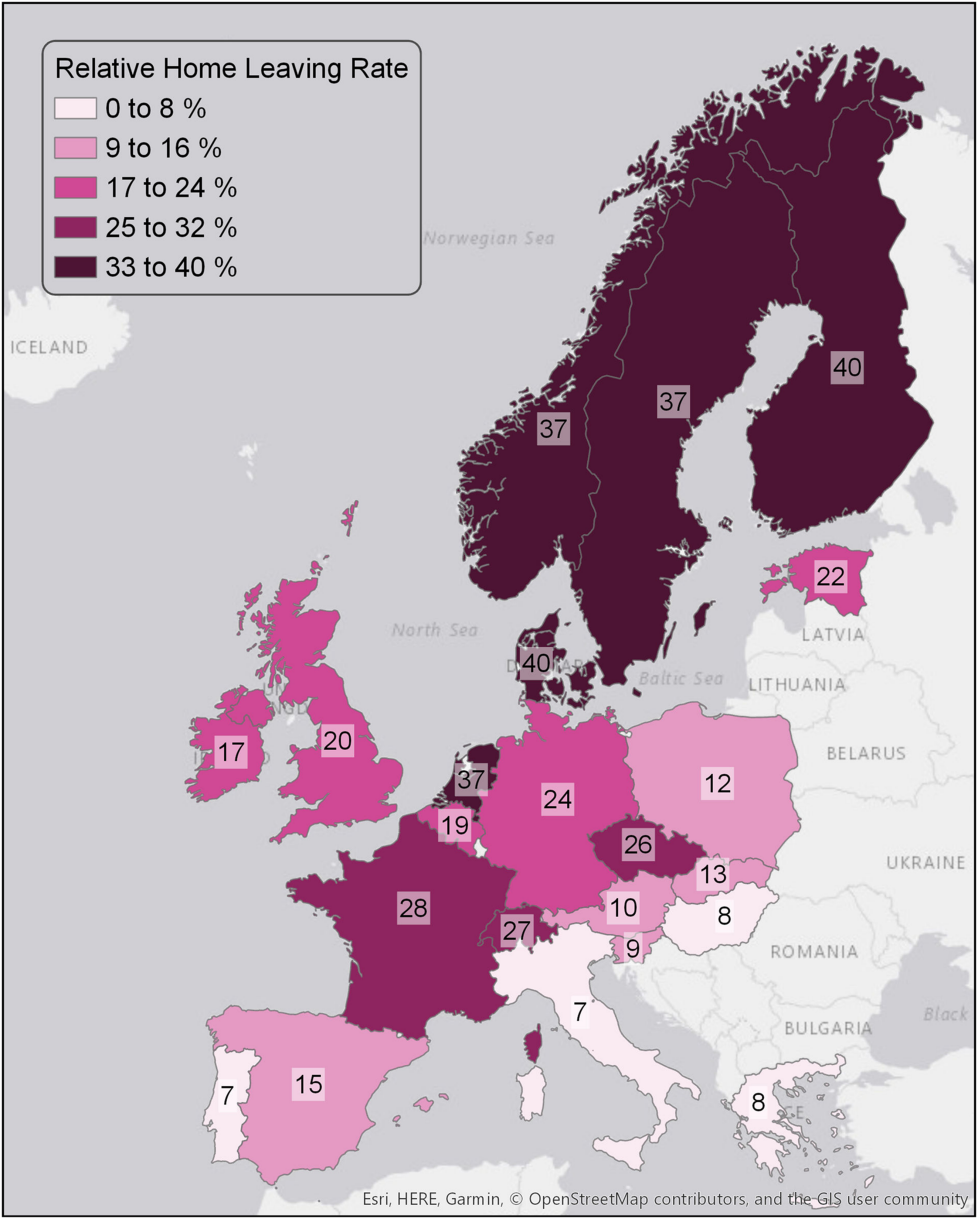
Relative home leaving rate of young adults aged 16 to 34 per country. Source: ESS 2002‐2016, own calculations

**FIGURE 2:**
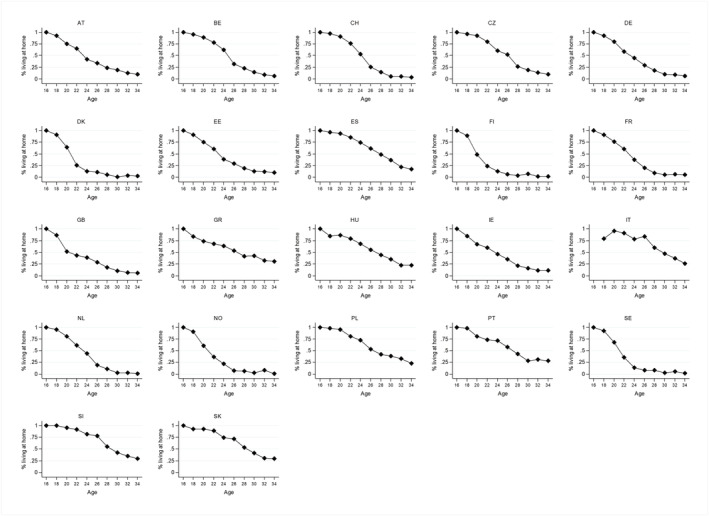
Percentage living at home per country. Source: ESS 2002‐2016, controlled for gender, own calculations

Figures 1 and 2 show the descriptive results per country for the relative home leaving rate (Figure 1) and the percentage living at home (Figure 2). These figures show the large country differences in leaving home, in particular a pronounced north–south divide. In general, home leaving is earlier and occurs at a higher rate in northern countries than in southern countries. Whereas, on average, 40% of the young adults who live at home in Finland and Denmark leave home between two waves, only 7% of the young adults who live at home in Italy and Portugal leave home between two waves. Moreover, home leaving occurs earlier and at a higher rate in Western Europe than in Eastern Europe. However, these geographical distinctions do not fit for all countries. For example, home leaving occurs relatively late and at a lower rate in Ireland and Belgium compared to other countries in these regions, whereas home leaving occurs relatively early and at a high rate in the Czech Republic. The geographical pattern for the home‐leaving rate in Figure 1 resembles the geographical pattern for the age of leaving home in the paper of Billari et al. (2001, p. 346). This suggests that our outcome variable based on the pseudo‐panel design captures what we seek to explain: country differences in leaving home.

**FIGURE 3:**
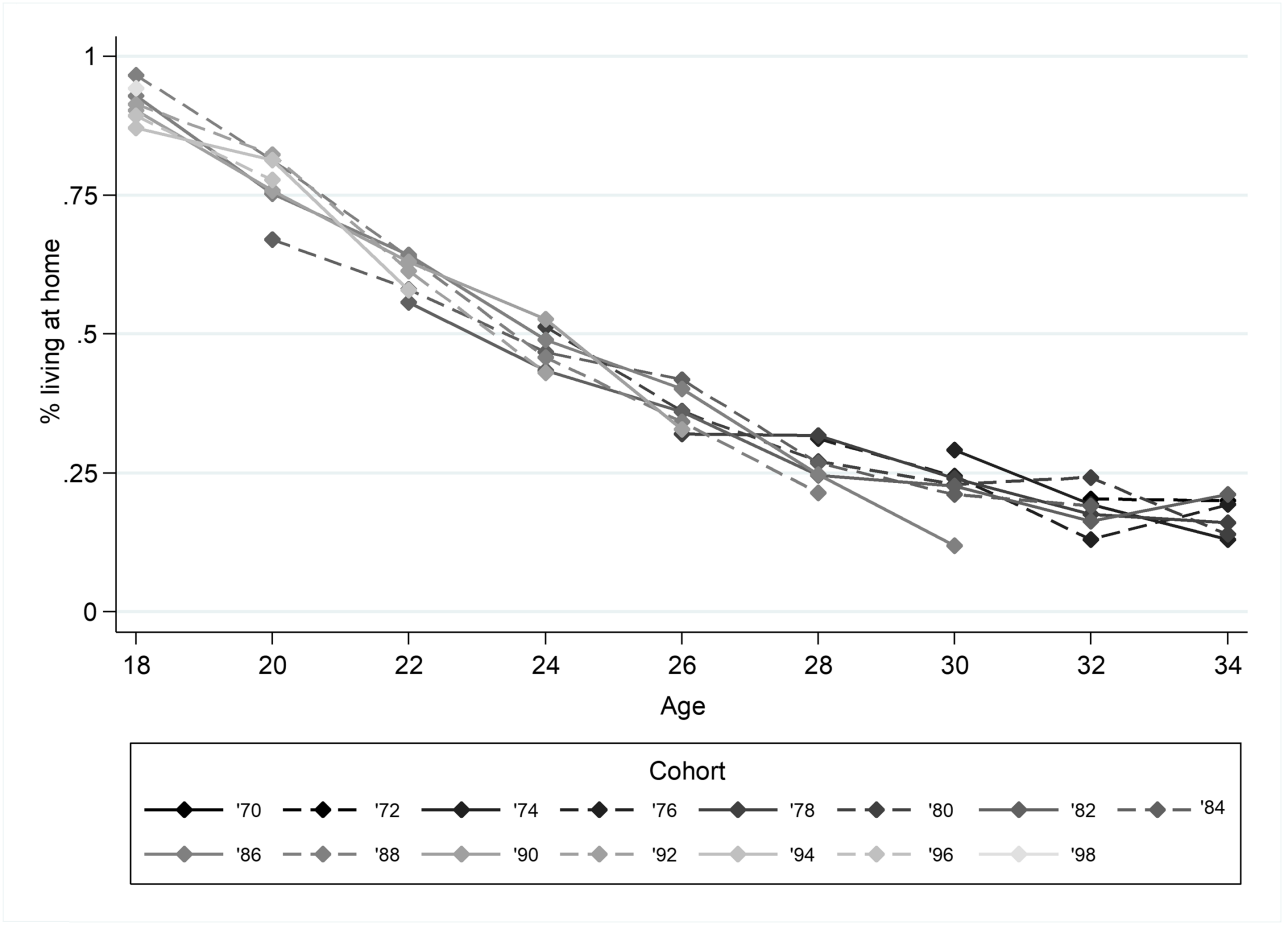
Percentage living at home per cohort. Source: ESS 2002‐2016, controlled for country and gender, own calculations

Figure 3 shows the percentage of young adults living at home at different ages per cohort. Note that for most cohorts only part of the age range is observed. The figure shows that the age of leaving home is comparable for the different cohorts. Our study is the first to compare home leaving for recent cohorts born between 1970 and 1999. Earlier research has reported an increase in the age of leaving home for the 1960s and 1970s cohorts compared to earlier cohorts (Billari & Liefbroer, 2010). Our findings suggest that this trend has stabilised in more recent cohorts.

### 4.2 Regression results

The results of the regression analyses on the effects of cultural, economic, and institutional characteristics on the relative home‐leaving rate are presented in Table 3. The coefficients in this table can be interpreted as percentage‐points changes in the relative home‐leaving rate for unit changes in the independent variables.

**TABLE 3:** Multilevel analysis on the relative home leaving rate

	Model 1	Model 2	Model 3	Model 4	Model 5
Year (ref. 2002–2004)					
2004–2006	−1.179 (2.642)	−1.478 (2.631)	−1.467 (3.154)	−1.377 (2.633)	−1.249 (3.082)
2006–2008	−0.202 (2.675)	−0.386 (2.675)	−0.800 (3.825)	−0.273 (2.668)	−0.411 (3.797)
2008–2010	−3.731 (2.553)	−3.674 (2.594)	−10.094** (3.813)	−2.557 (2.550)	−7.287 + (3.716)
2010–2012	−0.396 (2.625)	−0.615 (2.678)	2.096 (5.558)	1.880 (2.839)	6.622 (5.549)
2012–2014	−2.408 (2.667)	−2.898 (2.789)	−4.387 (4.827)	0.335 (2.860)	−1.223 (4.799)
2014–2016	2.409 (2.822)	2.035 (2.904)	−0.094 (4.893)	4.894 (2.977)	3.523 (4.834)
Age (centred)	2.122*** (0.144)	2.129 (0.144)	2.124*** (0.144)	2.128*** (0.144)	2.129*** (0.144)
Age squared (centred)	−0.177*** (0.030)	−0.177*** (0.030)	−0.179*** (0.030)	−0.177*** (0.030)	−0.179*** (0.030)
Male	−5.258*** (1.391)	−5.252*** (1.391)	−5.243*** (1.389)	−5.278*** (1.390)	−5.272*** (1.388)
Religiosity		−3.038 + (1.744)			−3.743* (1.498)
Individualistic values		7.767*** (1.729)			4.456 + (2.312)
Responsibilities parents		−1.032 (1.372)			−0.787 (1.271)
Youth unemployment			−0.597 (1.381)		1.448 (1.321)
Change in youth unemployment			−1.994 (1.065)		−3.040** (1.065)
GDP			5.828** (2.091)		2.431 (1.876)
Change in GDP			2.133 (1.725)		2.492 (1.746)
Housing prices			−1.177 (1.043)		−0.130 (1.028)
Change in housing prices			−0.604 (1.065)		−0.468 (1.085)
Net replacement rate				7.674*** (1.686)	3.668* (1.859)
Social spending as % of GDP				−1.877 (1.777)	−1.897 (1.646)
Constant	31.403*** (3.358)	32.098*** (2.619)	32.775*** (3.905)	31.506*** (2.830)	31.443*** (3.503)
σu (country)	149.712 (49.960)	47.174 (18.199)	83.847 (31.219)	76.580 (27.180)	26.003 (12.434)
σu (intercept)	923.313 (29.939)	923.956 (29.960)	920.491 (29.868)	921.722 (29.885)	919.370 (29.838)
ICC (country)	0.140	0.049	0.083	0.077	0.028

*Note*: All independent variables are standardised. Number of CCGY groups: 1925, number of countries: 22.

^*^ p < 0.05,

^**^ p < 0.01,

^***^ p < 0.001.

The first model includes only the control variables. In this model, the ICC is 0.140, indicating that 14% of the remaining variance in the relative home‐leaving rate between CCGY groups is at the country level. This is a large variance component showing heterogeneity across Europe in the timing of leaving home. The effects of the year variables are negative for changes from the first wave up to the penultimate wave and positive for the last wave. However, none of these estimates are statistically significant. This does not mean that the recession had no effect. It is difficult to periodize these effects across countries because the recession unfolded at a different pace and intensity across countries. The economic factors added in Model 3 are country‐specific reflections of the recession that give further insight. The results for the age terms show that the relative home‐leaving rate increases with age but the positive effect decreases with higher ages (negative age‐squared effect). Consistent with previous micro‐level research, the home‐leaving rate of men is lower than the rate of women.

In Model 2, we add the cultural indicators. There is a positive effect of individualistic family values on leaving home. With a one‐*SD* increase in this contextual variable, the mean of the relative home‐leaving rate is expected to increase by almost eight percentage points, indicating more home leaving in country‐periods with more individualistic values. Religiosity delays home leaving, but this effect is weaker and insignificant. The coefficient of the responsibility of parents points in the expected direction, indicating slower home leaving, but the effect is weak and insignificant. As the ICC drops to 5%, these three cultural indicators explain more than half (65%) of the cross‐national differences in leaving home. All in all, in line with Hypothesis 1 home leaving occurs earlier in individualistic than in familialistic countries and this explains a large share of the cross‐national variation in leaving home.

In Model 3, we examine the economic explanations for home leaving. We find a negative but insignificant main effect of the youth unemployment rate. However, the change in youth unemployment between two waves does affect leaving home. The relative home‐leaving rate is lower if there is an increase in youth unemployment. There is a positive significant effect of GDP. A one‐SD increase in GDP is associated with a six percentage points increase in the home‐leaving rate. The housing prices, change in housing prices, and the change in GDP are less important predictors of home leaving. In total, the economic indicators explain 41% of the cross‐national variation in the relative home‐leaving rate (ICC decline from 0.14 to 0.08). This supports our Hypothesis 2 which holds that young adults leave the parental home earlier in country‐periods with more favourable economic circumstances. Specifically, a lower GDP and increasing youth unemployment delay home leaving.

Model 4 includes the institutional factors. We find a strong positive effect of the NRR, which is the percentage of the wage that individuals receive after becoming unemployed. The relative home‐leaving rate increases by approximately 7.5 percentage points with a one‐SD increase in the NRR, indicating that an institutional safety net promotes home leaving. The partial effect of the social spending variable is negative but weak and insignificant. Taken together the institutional characteristics explain 45% of cross‐national variation in the relative home‐leaving rate. In line with Hypothesis 3, home leaving is earlier in country‐periods in which there are higher welfare state provisions.

**FIGURE 4:**
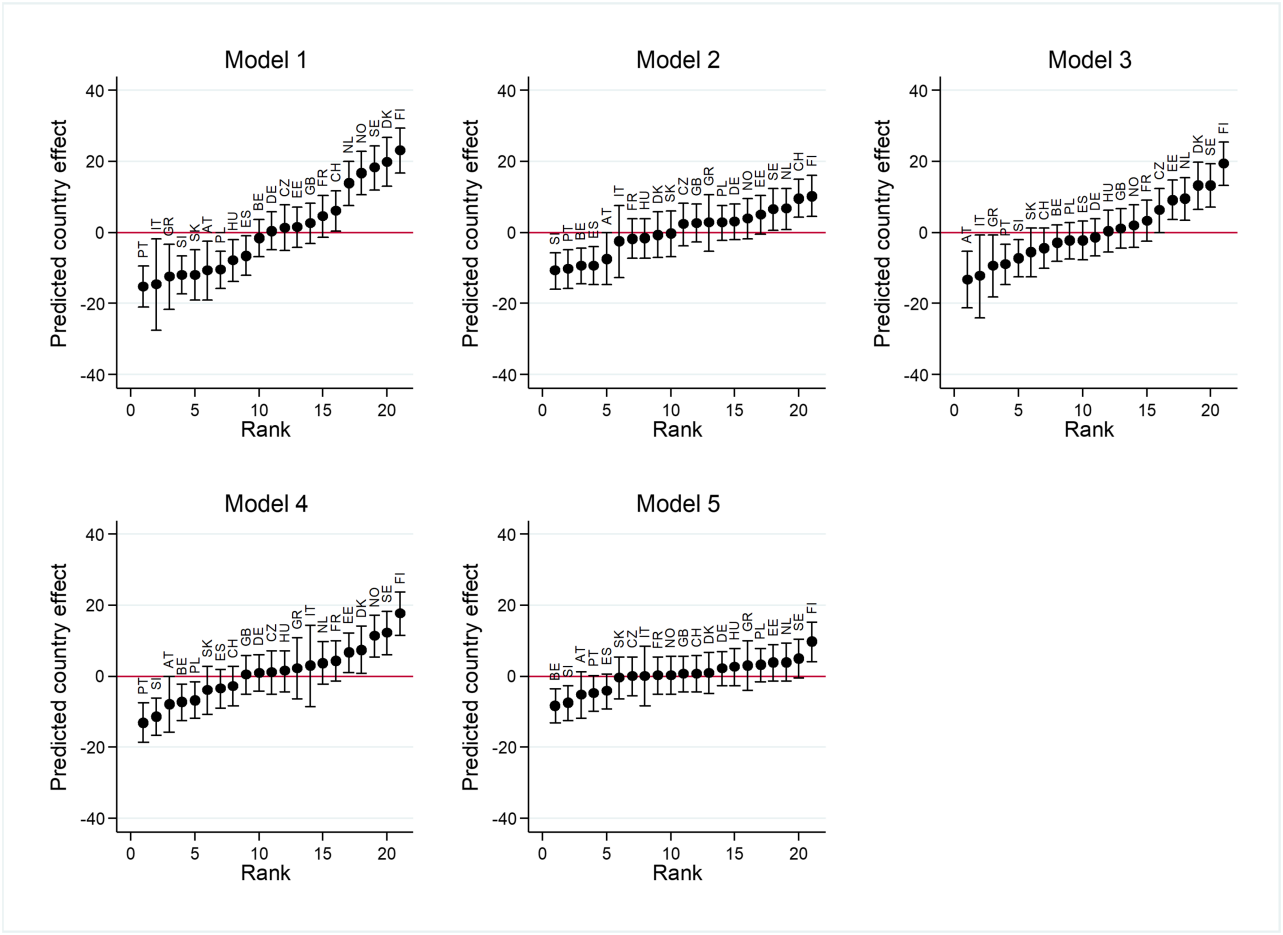
Predicted country effects in each of the models. Source: ESS 2002‐2016, own calculations

In Model 5, we include cultural, economic, and institutional characteristics simultaneously. In this model, the size of the effects of individualistic values, the NRR, and in particular the GDP decrease. Although some of the macro‐level characteristics are correlated, they also have additive effects. The ICC in the final model is 0.028, implying that 80% of the cross‐national variation is explained by our cultural, economic, and institutional indicators. Figure 4 illustrates how the variation between countries is reduced by a comparison of the predicted country effects in each of the models. We see that the models explain, for example, the low home‐leaving rate in Italy. Whereas Italy is at the right‐side of the y‐axis in Model 1 because of a lower than average home‐leaving rate, it is in the middle in Model 5. The figure shows that most of the low home‐leaving rate in Italy is explained by institutional characteristics. In general, the cultural indicators explain most of the cross‐national variation in home leaving. We find more support for this finding in an additional check of how much of the variance is explained if one of the sets of indicators is not included in the model. The highest share of the ICC is explained in the model in which the institutional indicators are excluded (77.1%) followed by the model excluding the economic indicators (70.7%), and the model excluding the cultural indicators (59.3%). Among the cultural indicators, the most important predictor is religion, followed by individualistic values, and responsibility of parents. Important structural indicators are GDP, change in unemployment, unemployment and the NRR.

Does the family or institutions function as a ‘safety net’ for poor economic conditions? We test whether the effects of the economic characteristics in a country‐year depend on the cultural or institutional setting in that country‐year by estimating interactions between the different contextual conditions. We only present interactions with the cultural, economic, and institutional characteristics that had a sizeable effect on the relative home‐leaving rate in the previous analyses. The results in Table 4 show that none of the interactions is significant, suggesting that there are no differences in the effect of economic characteristics in societies with different cultural or institutional characteristics. Hence, we have to reject Hypotheses 4 and 5.

**TABLE 4:** Multilevel analysis on the relative home leaving rate, culture and economic interactions

	Model 1	Model 2	Model 3	Model 4	Model 5	Model 6
Year dummies included	Yes	Yes	Yes	Yes	Yes	Yes
Religiosity	−5.794** (2.043)	−3.990 (2.043)				
Individualistic values			8.978*** (1.690)	7.197*** (1.964)		
Net replacement rate					7.819*** (1.521)	5.286** (1.769)
Change in youth unemployment	−2.107* (0.938)		−2.605** (0.987)		−3.466** (1.006)	
GDP		6.291*** (1.783)		3.076* (2.056)		4.355* (1.962)
Religiosity* change in youth unemployment	−0.737 (0.582)					
Religiosity * GDP		1.659 (1.667)				
Individualistic values * change in unemployment			−0.292 (0.834)			
Individualistic values * GDP				−0.585 (1.203)		
Net replacement rate * change in youth unemployment					−0.999 (0.792)	
Net replacement rate * GDP						−0.666 (1.797)
Constant	33.146*** (3.036)	36.653*** (3.109)	33.496*** (2.692)	34.097*** (3.054)	32.728*** (2.764)	34.306*** (3.222)
σu (country)	99.214 (34.851)	60.792 (25.520)	58.761 (21.186)	57.542 (21.452)	65.386 (23.241)	63.941 (23.028)
σu (intercept)	920.953 (29.868)	924.205 (30.020)	920.194 (29.828)	922.873 (29.925)	918.018 (29.758)	922.343 (29.902)
ICC (country)	0.097	0.062	0.060	0.059	0.066	0.065

*Note*: All independent variables are standardised. Controls for age, age squared and gender included. Number of CCGY groups: 1925, number of countries: 22.

^*^ p < 0.05,

^**^ p < 0.01,

^***^ p < 0.001.

### 4.3 Robustness analyses

As a robustness check, we replicated the analyses without removing the outliers. These analyses did not differ substantially from our main analyses. Additionally, we explored different modelling techniques to account for time or cohort effects. We use cross‐classified models by country and time or cohort, and three‐level multilevel models with country‐cohorts or country‐years nested in countries. These models suggest that there is little variation across time or cohorts, rendering these modelling strategies unnecessary. The main effects of these models are in line with our findings. Finally, we examined several other variables. In particular, we examined the effects of housing variables other than housing prices. Rental housing prices, housing costs, and homeownership rate did not show sizable effects on the home‐leaving rate. Moreover, we examined the effect of the percentage of young adults in education. We did this both specific per CCGY group and per country‐year group. Both analyses indicated no substantial effect, suggesting that education does not explain cross‐national differences in leaving home in Europe.

## 5 DISCUSSION

Motivated by the question of how cross‐national differences in the age of leaving home could be explained, we tested cultural, economic, and institutional explanations using pseudo‐panel data for 22 countries. In total, we were able to explain 80% of the cross‐national variation in Europe. More specifically, we have shown that home leaving is late in societies with lower levels of religiosity, more individualistic family values, a higher GDP, declining youth unemployment, and a higher net replacement rate.

Our findings speak to three debates in the literature on (youth) transitions. First, they contribute to the long‐standing debate about culture vis‐à‐vis structural explanations for both societal differences and long‐term trends in demographic behaviours (Bachrach, 2014; Blossfeld et al., 2006; Lesthaeghe, 2014; Lesthaeghe & Surkyn, 1988; Oppenheimer, 1988; Reher, 1998; Van De Kaa, 1987; Zaidi & Morgan, 2017). Our study shows that even when we account for a rich set of year‐specific measures of economic and institutional characteristics, cultural characteristics still explain a large share of cross‐national differences in leaving home. This finding supports research emphasising the role of the cultural context, such as work on the SDT (Lesthaeghe, 2007; Van De Kaa, 1987) and weak and strong family systems (Reher, 1998). We show that home leaving is not just the result of a rational decision‐making process that depends on economic and institutional constraints and opportunities (Bachrach, 2014). Whereas research on leaving home has typically aimed to explain differences in leaving home from this rational point of view ‐ especially on the micro‐level (e.g., Avery et al., 1992)—we argue that more attention should be paid to cultural values.

Second, our study contributes to the question whether the prolongation of the transition to adulthood in recent years is the result of more opportunities for young people to explore adult roles or of constraints that ‘arrest’ the transition to adulthood (Cuzzocrea, 2019; Woodman & Leccardi, 2015; Wyn, 2014). Although popular accounts on leaving home often refer to late home leavers as ‘mama's boys’ benefitting from ‘Hotel Mama’, our findings show that home leaving is partially late because of a lack of institutional support and poor economic conditions. This is an important insight for academic work on home leaving, as well as for policymakers who aim to support young adults' transitions to adulthood. Our findings suggest that recent increases in youth unemployment following the COVID‐19 pandemic may further delay young people's moves out of the house and their subsequent transition to adulthood.^iv^ Institutional support would be effective in supporting young adults' moves out of the home.

Third, our finding that the role of economic characteristics does not depend on the role of cultural or institutional characteristics, as suggested by a lack of significant interaction effects, contributes to questions about differences in the role of the family. While families in more familialistic countries with little state support are expected to be more likely to step in and offer housing in times of rising unemployment and GDP falls (Settersten, 2007), we find that this is not the case. This finding provides an insight for the role of family in times of need (Cuervo & Fu, 2020). Lockdowns and economic effects of the COVID‐19 pandemic may result in delayed home leaving in most countries and not only in countries with strong family ties and little state support.

A disadvantage of our pseudo‐panel design is that we were not able to capture micro‐level factors. As our macro‐level characteristics are able to explain most of the cross‐national variation, the prime aim for future research is not to look for new factors or better explanations but instead to adjudicate between these factors and to link their role to the micro‐level of analysis. In particular, it would be interesting to examine whether unemployment rates and institutional support mainly affect unemployed individuals or whether they affect individuals regardless of their employment status. If the former is the case, policy makers and researchers should pay more attention to that specific group and their ability to leave home. If the latter is the case, the focus should be on young people's uncertainties surrounding their future.

Our design also contributes to the literature on life‐course events. Compared to the co‐residence rate, which is generally used as an outcome variable in cross‐national research in this literature, our home‐leaving measure estimates more precisely when home leaving occurs. This allows us to see, for example, if home leaving is more common in times of poor economic conditions. Future research on other life course outcomes such as marriage and fertility could adopt similar designs and take a more dynamic approach to predicting cross‐national variation in demographic behaviour. The advantage of our design over a design based on a cross‐national comparison of the percentages of young people who have a child or are married is that our design captures the timing of the transitions. The advantage of our design over fertility or marriage rates is that our design takes the potential to experience a transition into account. For example, marriage rates may be low because a large share of the population is already married. By looking at relative rates, our design circumvents this issue.

## Data Availability

The data that support the findings of this study are openly available in the Norwegian Centre for Research Data, Norway Data Archive and distributor of ESS data for ESS ERIC. doi:10.21338/NSD‐ESS‐CUMULATIVE, European Social Survey Cumulative File, ESS 1‐9 (2020). Data file edition 1.0. NSD; GESIS data archive. doi:10.4232/1.14804, Integrated Values Surveys 1981–2015 (EVS Longitudinal Date File 1981–2008, ZA4804: v.2.0.0, 2011‐12‐30; World Values Survey: All Rounds Country‐Pooled Datafile Version. Madrid: JD Systems Institute.) World Bank. *World Development Indicators*, The World Bank Group, https://data.worldbank.org/. (Accessed April 2021) OECD (2021), Housing prices (indicator). doi: 10.1787/63008438‐en (Accessed April 2021); Social spending (indicator). doi: 10.1787/7497563b‐en (Accessed April 2021); Benefits in unemployment, share of previous income (indicator). doi: 10.1787/0cc0d0e5‐en (Accessed April 2021).

## ENDNOTES

i Based on calculations by the authors using the ESS round 9.

ii In light of little variation in this measure per country across the time period studies, we expect the use of interpolation techniques to be an appropriate solution for the lack of year‐specific data for this variable. On average, the variation in this measure per country is less than half a standard deviation between 1999 and 2008.

iii In 2006, the sampling method in Ireland changed from a method sampling individuals from the Electoral Register to sampling households from the GeoDirectory. The earlier sampling method led to an underestimation of single households and young adults living independently in the first two waves of the study.

iv
https://ec.europa.eu/eurostat/statisticsexplained/index.php/Unemployment_statistics#Youth_unemployment

## 1

**TABLE A1:** Percentage home leavers aged 15 to 35 who again live in the parental home

Country	Percentage
Czech	1,1
Austria	2,2
Finland	5,3
Great‐Britain	5,8
France	8,1
The Netherlands	11,0
Norway	11,3
Estonia	12,4
Ireland	12,8
Germany	13,2
Belgium	18,1
Switzerland	18,1
Hungary	20,0
Poland	21,5
Italy	24,7
Slovenia	25,3

**TABLE A2:** Sample selection process. Number of country‐year‐cohorts and countries

Step	Reason dropping cases	N level 1: country‐cohorts‐gender‐year groups (dropped)	N level 3: countries (dropped)
*Starting Sample*		2585	30
1. Drop if co‐residence percentage was <5% in the year before	Relative home leaving in these cases might not be too dependent on small sample differences	2334 (251)	30 (0)
2. Drop if one of the main indicators is missing		1983 (351)	22 (8)
3. Drop outliers		1961 (22)	22 (0)
4. Drop observations in Ireland between 2002 and 2006	The sampling method in the first two waves was individual‐based, whereas it was household‐based in the later waves.	1925 (36)	22 (0)
*Final sample*		1925	22

**TABLE A3:** Number of country‐cohorts‐gender groups per year

Cohort	2002–2004	2004–2006	2006–2008	2008–2010	2010–2012	2012–2014	2014–2016
1. 1970–1971	25						
2. 1972–1973	29	21					
3. 1974–1975	27	25	18				
4. 1976–1977	29	25	25	27			
5. 1978–1978	35	30	27	31	25		
6. 1980–1981	35	35	32	33	26	24	
7. 1982–1983	37	36	35	35	32	22	20
8. 1984–1985	36	35	34	36	31	25	15
9. 1986–1987	29	34	35	39	36	32	20
10. 1988–1989		34	34	39	38	34	28
11. 1990–1991			30	37	36	33	29
12. 1992–1993				38	36	34	30
13. 1994–1995					31	33	27
14. 1996–1997						33	28
15. 1998–1999							25
Total	282	275	270	315	291	270	222
